# Transcriptomics in Toxicogenomics, Part II: Preprocessing and Differential Expression Analysis for High Quality Data

**DOI:** 10.3390/nano10050903

**Published:** 2020-05-08

**Authors:** Antonio Federico, Angela Serra, My Kieu Ha, Pekka Kohonen, Jang-Sik Choi, Irene Liampa, Penny Nymark, Natasha Sanabria, Luca Cattelani, Michele Fratello, Pia Anneli Sofia Kinaret, Karolina Jagiello, Tomasz Puzyn, Georgia Melagraki, Mary Gulumian, Antreas Afantitis, Haralambos Sarimveis, Tae-Hyun Yoon, Roland Grafström, Dario Greco

**Affiliations:** 1 Faculty of Medicine and Health Technology, Tampere University, FI-33014 Tampere, Finland; antonio.federico@tuni.fi (A.F.); angela.serra@tuni.fi (A.S.); luca.cattelani@tuni.fi (L.C.); michele.fratello@tuni.fi (M.F.); pia.kinaret@helsinki.fi (P.A.S.K.); 2 BioMediTech Institute, Tampere University, FI-33014 Tampere, Finland; 3 Center for Next Generation Cytometry, Hanyang University, Seoul 04763, Korea; hakieumy12@gmail.com (M.K.H.); gksakdma0529@gmail.com (J.-S.C.); taeyoon@hanyang.ac.kr (T.-H.Y.); 4 Department of Chemistry, College of Natural Sciences, Hanyang University, Seoul 04763, Korea; 5 Institute of Next Generation Material Design, Hanyang University, Seoul 04763, Korea; 6 Institute of Environmental Medicine, Karolinska Institutet, 171 77 Stockholm, Sweden; pkpekka@gmail.com (P.K.); penny.nymark@ki.se (P.N.); grafstromrc@gmail.com (R.G.); 7 Division of Toxicology, Misvik Biology, 20520 Turku, Finland; 8 School of Chemical Engineering, National Technical University of Athens, 157 80 Athens, Greece; irini.liampa@gmail.com (I.L.); hsarimv@central.ntua.gr (H.S.); 9 National Institute for Occupational Health, Johannesburg 30333, South Africa; natashaS@nioh.ac.za (N.S.); maryG@nioh.ac.za (M.G.); 10 Institute of Biotechnology, University of Helsinki, 00014 Helsinki, Finland; 11 QSAR Lab Ltd., Aleja Grunwaldzka 190/102, 80-266 Gdansk, Poland; k.jagiello@qsarlab.com (K.J.); t.puzyn@qsarlab.com (T.P.); 12 Faculty of Chemistry, University of Gdansk, Wita Stwosza 63, 80-308 Gdansk, Poland; 13 Nanoinformatics Department, NovaMechanics Ltd., Nicosia 1065, Cyprus; melagraki@novamechanics.com (G.M.); afantitis@novamechanics.com (A.A.); 14 Haematology and Molecular Medicine Department, School of Pathology, University of the Witwatersrand, Johannesburg 2050, South Africa

**Keywords:** toxicogenomics, transcriptomics, RNA-Seq, scRNA-Seq, microarray, data preprocessing, quality check, normalization, batch effect, differential expression

## Abstract

Preprocessing of transcriptomics data plays a pivotal role in the development of toxicogenomics-driven tools for chemical toxicity assessment. The generation and exploitation of large volumes of molecular profiles, following an appropriate experimental design, allows the employment of toxicogenomics (TGx) approaches for a thorough characterisation of the mechanism of action (MOA) of different compounds. To date, a plethora of data preprocessing methodologies have been suggested. However, in most cases, building the optimal analytical workflow is not straightforward. A careful selection of the right tools must be carried out, since it will affect the downstream analyses and modelling approaches. Transcriptomics data preprocessing spans across multiple steps such as quality check, filtering, normalization, batch effect detection and correction. Currently, there is a lack of standard guidelines for data preprocessing in the TGx field. Defining the optimal tools and procedures to be employed in the transcriptomics data preprocessing will lead to the generation of homogeneous and unbiased data, allowing the development of more reliable, robust and accurate predictive models. In this review, we outline methods for the preprocessing of three main transcriptomic technologies including microarray, bulk RNA-Sequencing (RNA-Seq), and single cell RNA-Sequencing (scRNA-Seq). Moreover, we discuss the most common methods for the identification of differentially expressed genes and to perform a functional enrichment analysis. This review is the second part of a three-article series on Transcriptomics in Toxicogenomics.

## 1. Introduction

The development of omic sciences gave unprecedented insights into physiological and pathological mechanisms at a molecular level, arguably in almost all the areas of life sciences, including toxicology [1]. The technological advances in the post-genomic era allowed the rise of toxicogenomics as an effective complementary approach in modern toxicology. Among all, the most employed omics technique in toxicology is undoubtedly transcriptomics, which allows the deep profiling of the transcriptome of a number of tissues and cell lines [2]. The hybridization-based technologies first, such as DNA microarrays, and the sequencing-based approaches, like RNA-Sequencing (RNA-Seq) later, conquered the market in the last two decades, achieving, nowadays, the resolution of the single cell.

The production of large data sets from transcriptomics experiments encouraged the birth of high-throughput studies, led by big consortia, aimed at generating public repositories that contain humongous amounts of gene expression data, which have been made available to the scientific community [3,4,5,6,7,8,9]. These rich sources of data are aimed not only to the identification of biomarkers of toxicity, but also to determine the link between their expression signatures to the toxicological phenotype of the organism for a particular exposure or dose and at a particular time, in order to satisfy the principle of “phenotypic anchoring” [1].

However, to carry out a rigorous analysis and obtain reliable results, a correct analytical procedure should be employed. Despite the well defined pipelines for generic transcriptome data analysis have been already designed [10], well established guidelines for the analysis of gene expression in a toxicogenomics setting have not been formulated. In fact, even a single transcriptomics experiment produces massive amounts of data, whose preprocessing and management are not straightforward [11]. Every transcriptomics experimental scenario could potentially have different optimal methods for transcript quantification, normalization, detection of surrogate variables and, ultimately, differential expression analysis. In addition, quality control checks should be applied pertinently at different stages of the analysis to ensure the reliability of the results. For all of the steps, a balance between the most updated and widely employed methods should be achieved. Moreover, keeping track of the statistical methods employed in the data preprocessing improves the reproducibility and the transparency of the analyses and, therefore, makes the data interpretation trustworthy [12,13].

In this work, we outline current standards, available resources and good practices for the bioinformatics analysis of transcriptomics data in toxicogenomics, generated from both microarray and RNA-Seq technologies. We discuss the new opportunities and challenges provided by single-cell RNA-seq and the analytical differences with the bulk RNA-Seq. Finally, we cover the most used algorithms for differential expression analysis and to perform a robust functional annotation, in order to elucidate the toxicity-related cellular processes.

## 2. Data Preprocessing

### 2.1. Microarray Experiments

The hypothesis underlying a microarray analysis is that relative gene expression levels are represented as fluorescence intensities. Indeed, microarray experiments allow investigating relationships between biological samples based on expression patterns. Thus, biologically relevant patterns are identified by investigating each gene expression ratio between different conditions [14]. However, this comparison cannot be performed before a number of transformations are carried out on the data to eliminate low-intensity measurements, to adjust the intensity values to perform robust comparisons and to identify differentially expressed genes.

**Figure 1:**
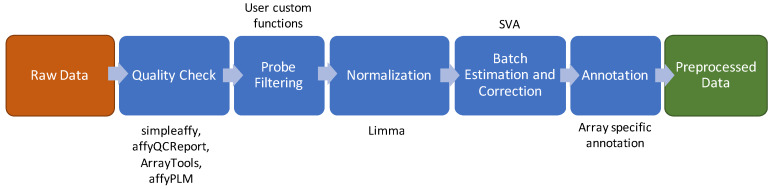
Data preprocessing schema for microarray. The brown box indicates the input of the pipeline. The green box indicates the output of the pipeline. The blue boxes show the intermediate steps of the pipeline and above or below the boxes are listed the software/packages employed in the step.

The current standard of a microarray data preprocessing pipeline is shown in Figure 1 and comprises the following steps: quality check, probe prefiltering, normalization, batch effect and surrogate variables estimation and correction [15]. A complete list of all the methods and R packages used to implement this pipeline is shown in Table S1.

### 2.2. Quality Check

The gene expression patterns measured from microarray experiments can be significantly affected by different sources of systematic and random errors that may occur at different levels of the experiment [16]. For example, an inappropriate experimental design may affect the data set as a whole; poor probe design or probe misannotation will affect the readout of a particular probe; sample mislabelling or inappropriate sample treatment may result in individual outlier arrays. Checking the quality of the samples before performing any kind of analysis is crucial. Quality check (QC) methods aim at homogenizing the shape of gene expression distributions and to increase the robustness of probe intensity measures across different samples. A particular care in the QC of microarray data should be posed to the detection of RNA degradation signals. A commonly employed procedure of quality check, which takes into account the RNA degradation level, is to compute the Normalized Unscaled Standard Error (NUSE) [17], the Relative Log Expression (RLE) [17] and the slope of the RNA degradation curve (RNADeg) [18]. The samples outlierness can be computed by investigating the distributions of the values of the three metrics. A sample could be considered an outlier by using a consensus on the three scores, giving particular relevance to the RNADeg value. The distributions of the values of these three metrics can be investigated by means of boxplot and the sample outlierness is evaluated for each measure based on the data distribution. Eventually, a concordance outlierness score was computed across the three metrics. In particular, a sample was removed from the analysis if considered an outlier in at least two out of three metrics, one of them being the RNA degradation curve. Overall, several quality check methods have been proposed and often based on visual inspection of the data [19]. See Table S2 for a full list of QC plots and libraries employed in this step.

Another invaluable form of quality assurance that is specific for toxicological applications, as well as assessing the effects of engineered nanomaterials (ENMs) on RNA levels, is the use of spike-in probes, in order to account for variation between the arrays. Typically, spike-in kits consist of a mixture of multiple positive control transcripts at known concentrations which, for instance, anneal to complementary probes on the microarray. Therefore, the design of the assay is able to control for any abnormalities in the labelling and hybridisation procedure including the potential interference by ENMs, which may occur on fluorescent dyes in use [20].

### 2.3. Probe Prefiltering

Microarray data commonly show a large number of probes in the background intensity range. Usually, these probes’ intensities do not show a marked variability across arrays. Hence, they combine a low variance with low intensity. Thus, in many cases, they might be detected as differentially expressed, although they are barely above the “detection” limit and are not very informative in general. For these reasons, such probes are filtered out prior to further preprocessing steps.

### 2.4. Normalization

Normalization plays an important role in the microarray preprocessing since it allows to adjust the individual hybridization intensities in order to perform meaningful biological comparisons [14]. In this context, different methods have been proposed [14,21,22] that adjust the distributions of the values. A common strategy is the scale normalization approach [21,23] that forces the different samples to have the same median absolute deviation. This strategy does not consider that the shape of the distributions of the different arrays may vary between each other, thus it might be less efficient. A different approach normalizes the samples by adjusting their variability, such as the Locally Weighted Scatterplot Smoothing (LOWESS) algorithm [21,24]. It has been proposed in order to remove the bias present in the data which most commonly shows a deviation from zero for low-intensity spots. Furthermore, one commonly used adjustment approach is the quantile normalization [25,26] that assumes the statistical distribution of each sample to be the same, hence applying a scaling approach that also accounts for the variability.

### 2.5. Batch Effect Estimation and Correction

Gene expression data coming from microarray experiments can be affected by non-biological variables. The variability in the gene expression values due to these types of variables is known as batch effect. Batch effects can arise for multiple reasons, such as ambient conditions during the sample preparation and handling, amplification, labelling, hybridization protocol, different sites/laboratories in which the experiments are performed, different chip or platform types and different scanners [27]. These batches have a detrimental effect on the quality of the data and can ultimately lead to incorrect results [28].

**Figure 2:**
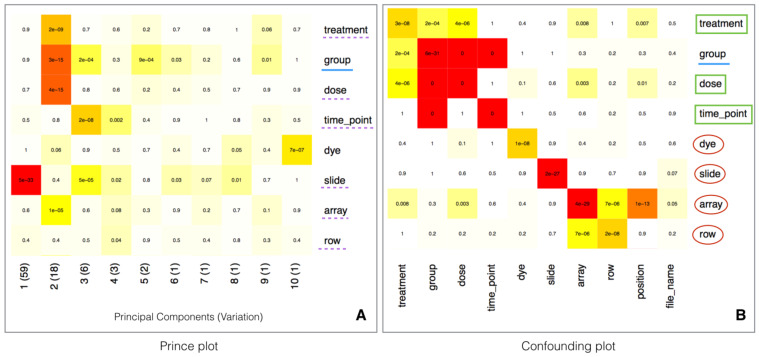
Panel **A**—Prince plot showing the association between the technical variables and the principal components. The text and the background color in each cell represent the association *p*-value. The row label underlined with solid blue line represents the variable of interest. The row labels underlined with dotted purple line represent other sources of high variation. Panel **B**—Confounding plot, representing the correlation among the technical variables. The row label underlined with solid blue line represents the variable of interest. The green squares represent the variables confounded with the variable of interest or other batch variables. The row labels circled by red outline are batch variables suitable for correction.

A fundamental step in the analysis is to attenuate the effects associated with batch variables while retaining the variation associated with biological variables. As previously discussed, to be able to properly correct the batch effects, the experimental design, including sample randomization and proper metadata annotation, needs to be carefully considered. Batch estimation and correction can be performed by using the surrogate variable analysis (SVA) R library [29]. Known biological (e.g., treatment, disease status, age, tissue) and technical (e.g., dye, array) variables are in general provided by the user in the phenotypic information, while unknown sources of variation can also be identified through the surrogate variable analysis [29]. The impact of the technical variables on the expression values can be identified by means of the prince plot (Figure 2A), that shows the correlation between the variables and the principal components of the expression matrix. In fact, the prince plot is a visualization of the output of Principal Components Analysis (PCA), which is a popular and powerful method to quantify the effect of batch variables, as well as to reveal the presence of unaddressed sources of batch behaviour in the data [30]. Moreover, the correlation between both biological and technical variables can be visually evaluated by the confounding plot (Figure 2B). This information is used to identify batch variables as known technical or surrogate variables which are associated with strong sources of variation and are not correlated with biological variables of interest.

These identified batch variables can be corrected to remove technical noise from the data. The R ComBat function [31], from the SVA package, can be used to remove the known batch variables and the estimated surrogate variables when not confounded with the variables of interest. Briefly, ComBat employs an empirical Bayes approach to estimate systemic batch biases affecting large sets of genes. The batch correction is carried out by specifying the variable of interest, any biological covariates, and a set of known batches or surrogate variables (obtained from the SVA, as described above). Since each run of ComBat function can only address the effect of one batch variable, any additional variables that cause known batch effects can be added directly to the linear model implemented for the differential expression analysis. When using SVA, it is important to have in mind that it will clear out the effect of any biological information that is not addressed by the known phenotype-related variables, such as phenotypic subgroups, that might be of interest [24]. For this reason, one can opt to use another linear modelling solution, for example the limma R package, that also permits the investigation of the effect of the covariates that are included in the model [32].

### 2.6. Probe Annotation

Accurate mapping of the microarray probes to genomic elements, such as genes or regulatory regions, is essential to generate reliable biological findings. However, the manufacturers of microarray platforms typically provide incomplete and/or outdated probe annotations, which often rely on older reference genome and transcriptome versions that differ substantially from up-to-date sequence databases [33]. To deal with these drawbacks, annotation pipeline tools have been proposed such as Re-annotator30. Annotations can also address conversion to different types of gene identifiers directly, such as Ensembl gene ID or Entrez gene ID. Furthermore, databases containing up-to-date annotation mapping are available, such as the Brainarray website (http://brainarray.mbni.med.umich.edu) from which the custom CDF file can be downloaded and used in combination with Bioconductor libraries to annotate Affymetrix microarray data.

### 2.7. Tools for Microarray Data Analysis

A huge collection of computational tools is available, both on CRAN and Bioconductor [34], to process omics data. However, the use of these tools requires a deep understanding of the statistics and methodological implementation. The integration of these tools as a unique workflow, requires proficiency in computer programming languages. Thus, a set of tools with graphical interface were developed to facilitate the analysis for the user such as AGA [35], shinyMethyl, MeV [36], O-miner [37], Chipster [38], Babelomics [39] and eUTOPIA [40]. Among all, eUTOPIA is the only one that implements all the steps of the microarray data preprocessing. In particular, all of the aforementioned tools implement the normalization steps, but some of them do not implement the quality check and probe filtering steps, and, more importantly, eUTOPIA and Chipster also allows to perform the batch effect estimation and correction, that is of extreme importance for microarray analysis because it can help to isolate technical noise from the biological signal.

## 3. RNA Sequencing

**Figure 3:**
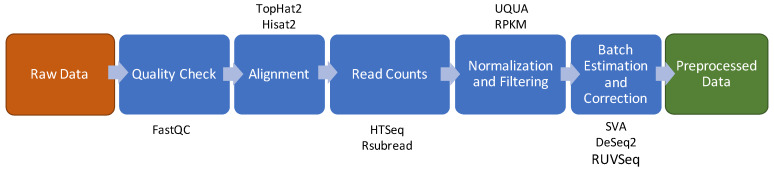
Data preprocessing schema for RNA-Seq. The brown box indicates the input of the pipeline. The green box indicates the output of the pipeline. The blue boxes show the intermediate steps of the pipeline and above or below the boxes are shown the software/packages employed in the step.

The state of the art of a typical RNA-Seq preprocessing pipeline is shown in Figure 3 and comprises the following steps: quality check, reads alignment, raw counts extraction, counts normalization and filtering and batch effect estimation and correction [10]. A complete list of all the methods and R packages used to implement this pipeline is shown in Table S3.

### 3.1. Quality Check

Similarly to microarray experiments, deep sequencing procedures may suffer from certain biases, which should be detected and corrected through an accurate quality check prior to subsequent analyses. From an experimental point of view, a pre-analitical check of the quality of the extracted RNA is necessary. There is currently no consensus to establish whether a sample is unusable based on the levels of RNA degradation. Thus, while standardized RNA quality metrics such as the Degradometer [41] or the RNA Integrity Number (RIN) [42], provide well-defined empirical methods to assess and compare sample quality, there is no widely accepted criterion for sample inclusion. First, the most common approach is to exclude from further analyses RNA samples with evidence of substantial degradation; this approach relies on establishing an arbitrary cut-off value for establishing the samples’ quality. Second, in case the decay of RNA is comparable, variation in gene expression estimates could be corrected by applying standard normalization procedures. Third, if transcripts decay at different rates, and if these rates are consistent across samples for a given level of RNA degradation, a model that takes into consideration measured, sample-specific, degradation levels could be applied to gene expression data to correct for the confounding effects of degradation [43].

Therefore, the first step for an accurate analysis of sequencing-based transcriptomics data is the quality check of the raw reads. This step is necessary in order to highlight biases and/or library contamination possibly occurred during the library preparation or sequencing procedure. One of the most widespread software to perform a quality check of the raw reads is FastQC (https://www.bioinformatics.babraham.ac.uk/projects/fastqc/). Such software is nowadays employed in the analysis of data obtained by Illumina platforms, but it was previously used also to analyse data produced from Roche 454 and Solid platforms. FastQC allows multi-sample analysis and provides a user-friendly and easy-to-use graphical interface. Beyond a general overview of the analysed sets of reads, reporting information like the number of reads in the considered file, their length and percentage of GC dinucleotides, the software shows the per-base quality of the entire set of reads. The quality is measured through the Phred score, expressed by the following formula:

$$q=-10\times \log_{10}\left(p\right)$$

where *p* indicates the error base-calling probability [44]. In general, if the Phred score of the 3’ bases of the reads is beyond a certain threshold (typically 20), it is a good practice to “trim” the reads in order to achieve an acceptable quality along all the sequence. The most common reads trimmers are Cutadapt [45], TrimGalore (http://www.bioinformatics.babraham.ac.uk/projects/trim_galore/) and the FASTX toolkit (http://hannonlab.cshl.edu/fastx_toolkit/). Such software can be used to both remove residual adapters used in the library construction and to trim the low-quality bases. Furthermore, FastQC provides information about the presence of contamination of the samples (e.g., unbalanced GC content in respect of the organism under study), presence of non-detected bases along with the reads, and overrepresented sequences.

### 3.2. Reads Alignment

The subsequent step is the alignment of the RNA-Seq reads onto a reference genome, in order to assign each of the sequenced reads to a specific location on the genome/transcriptome. This procedure is extremely challenging from a computational point of view. First, a number of reads that spans between a few millions to hundred millions undergoes alignment on a reference genome, whose dimension, in human, is around 3 billions base pairs. Moreover, since transcripts in eukaryotic genomes contain not contiguous regions (e.g., introns), alignment tools for RNA-Seq reads should handle spliced alignment with very large gaps. Therefore, Trapnell and colleagues [46] developed a splice-aware algorithm able to align sequencing reads to a reference genome accurately, in a reasonable time and without relying on known splice sites. This algorithm revolutionized the way of mapping sequencing reads to a genome since it was the first one able to detect de novo splicing junctions, aligning reads towards non-contiguous regions of the genome [47]. This feature boosted the discovery of non-annotated transcripts and novel splicing isoforms. Later, since the technological development led to the production of longer and paired reads, the same research group reported several improvements to the TopHat algorithm, developing TopHat2 [48]. Specifically, TopHat2 maps the reads in two steps: (1) the software aligns first the reads toward a reference transcriptome and (2) it maps the unmapped reads from the first step to the entire reference genome. This approach not only allows to improve the reads mapping over small insertions and deletions, but also to deal in a better way with processed pseudogenes. In addition, TopHat2 implements a fusion-detecting algorithm able to detect fusion transcripts deriving from translocation events and from big insertions and deletions. Meanwhile, the sequencing throughput and read lengths have significantly increased to several million reads per sample with lengths of hundreds of base pairs. More recently, Pertea and colleagues, developed the so-called “Tuxedo 2” pipeline [49], which fully addresses the challenges posed by the current technologies. The Tuxedo 2 pipeline integrates reliable software which allows a reads’ mapping step, transcript assembling and differential expression analysis. To map paired-end reads which are long at least 75–100 bp, we suggest using the HISAT2 algorithm. Briefly, the HISAT2 developers designed an efficient and innovative indexing method of the genome, an extension of the well-known Burrow-Wheeler Transform (BWT), named Graph-based FM index (GFM) which ideally allows to align sequencing reads against a genome representative of the general human population as well as against a single reference genome. Additionally, HISAT2 utilizes a large set of local indexes covering genomic regions of 56 kilobases (https://ccb.jhu.edu/software/hisat2/index.shtml). This novel approach may be definitely considered a current gold standard in the processing of the Next Generation Sequencing (NGS) reads in current and upcoming toxicogenomics experiments.

### 3.3. Raw Counts Extraction

Once the read mapping is accomplished, it is crucial to assign the mapped reads to certain genomic features (i.e., genes or exons). Since this task is aimed at the quantification of gene/transcript wise expression, the choice of the annotation is important. Nowadays, plenty of different annotations, produced by different consortia, are available. Such annotations are rapidly evolving as novel transcripts and splice isoforms are discovered and validated [50]. As a consequence, the user may find the choice of a proper annotation quite dispersive. In fact, based on the specific needings of the user, one may have to choose between narrow but well-curated annotations as well as the NCBI Reference Sequence collection (RefSeq [51]). RefSeq provides a reliable genomic annotation, continuously curated from the staff that includes automated computational methods, collaboration, and manual data review. On the other hand, Ensembl (http://www.ensembl.org) and GENCODE (https://www.gencodegenes.org) provide more comprehensive and updated annotations. Ensembl includes automatically annotated entries, while GENCODE derives from merging the Ensembl automatically annotated entries and the manually curated entries reported by the HAVANA team of the Wellcome Trust Sanger Institute from the Vega database [52].

Since the transcriptome analysis in a toxicogenomics setting is aimed at the study of gene expression deregulation upon the exposure of a certain compound on a biological system, a correct quantification of gene expression is crucial. In this step, the aligned raw reads are summarized into a count matrix which can be used for differential expression analysis [53]. The count matrix usually reports genes (or more in general the genomic features of interest) in rows and samples in columns. HTSeq [54] is among the most utilized algorithms developed until now for gene and transcript expression quantification. HTSeq is a package developed in Python language which offers a suite of functionalities for the parsing and the analysis of high-throughput sequencing data. However, the construction of reproducible and solid analysis workflows often imposes the researchers to consistently perform all the steps in the R environment. A clever solution for this restraint could be the employment of the Bioconductor Rsubread package [53]. This package implements functions for several steps of the preprocessing of NGS reads, as well as the alignment and reads summarization. Hereby, we suggest using it, especially for this second functionality, through the featureCounts function. Moreover, for more specific purposes, Rsubread allows the summarization of the reads by exon and splice junction rather than by gene, in order to inspect the exon usage and, for instance, alternative splicing [53].

### 3.4. Normalization and Filtering

In order to carry out a reliable downstream analysis, as well as differential expression and functional annotation of transcriptome data, the samples need to undergo normalization and filtering steps. The former is needed since the transcript quantification step is directly dependent on the length of the transcripts and the library size, in order to make comparable (1) the expression levels among the transcripts of the same sample, (2) the sequenced samples between each other. The latter is aimed at removing the low read counts since they correspond to the irrelevant biological features. Regarding the normalization of RNA-Seq data, the classical and most widespread method is the Reads per Kilobase of exon model per Million mapped reads (RPKM) [55]. This method performs a double-step normalization. In fact, it allows a “within sample” normalization, scaling the read count value of each transcript on the base of the length (expressed in kilobases) of that transcript. At the same time, the RPKM method performs a “between samples” normalization correcting the read counts on the base of the library size [56]. For the paired-end reads, the algorithm takes the name of Fragments per Kilobase of exon model per Million mapped reads (FPKM) since it considers the fragment (both pairs) rather than the single read. Although many other read counts normalization methods arose in the last years, the RPKM/FPKM method is still one of the most largely employed in transcriptomics. In 2010, Bullard and colleagues demonstrated that the differential expression evaluated after a per-lane normalization (as for RPKM/FPKM) may be heavily biased by a small proportion of highly expressed features. To overcome this limitation, they proposed a new normalization method [57] which scales the read counts by the upper quantile of the counts distribution (UQUA), after filtering out the genes whose read counts are significantly low in all the samples. In fact, after the read counts have been normalized and made comparable across samples, it’s important to filter out the low or zero read counts. Genes which are not expressed in any of the analysed conditions not only generate an uninformative signal but also weaken the sensitivity in differentially expressed genes detection. By filtering low counts genes, will enrich for true differential expression while simultaneously reducing the number of hypotheses tested, making, as a consequence, multiple testing adjustment less severe [58]. Therefore, we strongly suggest to filter out the low (or non-) expressed features prior testing for differential expression in order to achieve a more robust statistical significance.

### 3.5. Batch Effect Estimation and Correction

As for hybridization based experiments, also high-throughput sequencing experiments may suffer from non-biological sources of variation. As already mentioned for the microarrays, Principal Component Analysis (PCA) can be a precious instrument in order to identify the features affected by batch surrogates. Principal components are able to capture both biological and technical variability and, when estimated after the biological variables have been taken into account, it is able to quantify the effects of artefacts in the data [30]. The above mentioned SVA Bioconductor package can be used to identify and estimate surrogate variables for unknown sources of variation [29] also in NGS experiments. In particular, the package SVA implements the svaseq function, which takes into account the different statistical distribution of NGS data in respect of microarrays. However, the widely used R packages edgeR [59] and DESeq2 [60] allow the user to correct for known unwanted variation by including the batch variables in the design formula. In order to detect and remove unwanted variation from high-throughput sequencing experiments, Risso and colleagues developed a method named RUVSeq [61], which allows to normalize the read counts and to adjust for nuisance technical effects at the same time. This method has been included in the homonym Bioconductor package and it implements some strategies already employed for the batch effect removal in microarray [62,63,64]. Specifically, RUVSeq can employ three different approaches in order to normalize the data and identify the factors of unwanted variation: (1) it can use negative control genes, such as genes which do not vary across the samples on the base of the biological conditions of interest, or (2) negative control samples for which the covariates of interest are constant; yet, (3) it can use residuals from a GLM analysis performed on the unnormalized counts. Therefore, we suggest employing this counts’ normalization strategy, especially in the cases where the unwanted variation factors are unknown.

## 4. Single Cell RNA-seq

**Figure 4:**
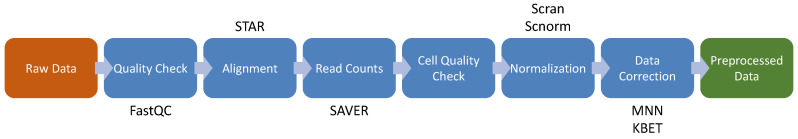
Data preprocessing schema for single-cell RNA-Seq. The brown box indicates the input of the pipeline. The green box indicates the output of the pipeline. The blue boxes show the intermediate steps of the pipeline and above or below the boxes are shown the software/packages employed in the step.

scRNA-seq technologies became more widespread and provided unprecedented opportunities for exploring gene expression profiles at single cell resolution, which greatly revolutionizes transcriptomic studies. Since the first publication about scRNA-seq methodology in 2009 [65], a number of scRNA-seq data analysis tools have been developed [66], that are available as scRNA-tools database (www.scRNA-tools.org) [67]. Nonetheless, the golden standard pipelines have not yet been established due to the technical noise, biological variation, the growing number of analysis methods and exploding data set sizes. Although some pipelines, such as Cell Ranger [68], inDrops [69], SEQC [70] and zUMIs [71] were proposed for the analysis of scRNA-seq data, they remain unexploited in most of the cases. In the sections below, we will discuss currently available methods for data preprocessing and analysis of scRNA-seq data. The common pipeline of scRNA-seq preprocessing is shown in Figure 4, which comprises the following steps: quality check on raw sequencing data, alignment, read counts extraction, cell quality check, normalization and data correction.

Since both bulk RNA-seq and scRNA-seq generally sequence transcripts into reads to generate the raw data in .fastq format, and the scRNA-seq data are often structurally identical to bulk RNA-seq data, the principles and methods used for data preprocessing of bulk RNA-seq, perhaps slightly modified, can be employed in most of the steps of scRNA-seq data preprocessing. For this reason, we will focus on analytical procedures which are specific for scRNA-Seq.

In scRNA-seq, one of the key challenges is to identify and remove low-quality cells that are damaged, dead or mixed with multiple cells. Typically, cell-level QC metrics are used to remove problematic cells. After cell QC, normalization is carried out for accurate comparisons of a gene’s expression across samples. Normalization methods developed for bulk RNA-seq are often used for scRNA-seq data. However, their suitability with respect to scRNA-seq data is still unexplored. For normalization of scRNA-seq data, several methods such as SCnorm [72] and Scran [73] have been recently proposed. SCnorm uses quantile regression to estimate the dependence of read counts on sequencing depth for every gene. Scran uses the summed expression values across pools of cells to conduct normalization. Log(x+1) transformation was recommended to examine log-fold changes in expression, mitigate the mean-variance relationship and reduce the skewness of the data after normalization.

Among steps in the data preprocessing pipeline for scRNA-seq, data correction aims to remove technical, biological and batch effects. The technical and biological effects can be regressed out by a simple or variant of regression model. Several methods to mitigate the batch effects in scRNA-seq data, such as Mutual Nearest Neighbor (MNN) and K-nearest neighbor Batch Effect Test (KBET), were proposed. From a recent comparison of classical batch effect correction methods [74], Combat [31] was confirmed to perform well. After read counts (also called expression quantification), digital gene expression matrices have the dimension of the number of barcodes (cell) multiplied by the number of transcripts (gene). The raw expression matrices often include over 20,000 genes. Because scRNA-seq experiments generate a portion of low-quality data from damaged or dead cells, cell quality check (QC) must be performed to ensure all cellular barcode data correspond to viable cells before downstream analysis.

### 4.1. Cell Quality Check

To exclude the low-quality data, which makes the downstream analysis difficult and may lead to misinterpretation, a series of QC analyses is required to ensure that the data quality is sufficient for downstream analysis. Three covariates are used for the QC: the number of counts per barcode (count depth), the number of genes per barcode, and the fraction of counts from mitochondrial genes per barcode [75,76]. The low-quality cells whose membranes are broken or doublets are discarded by examination of the distribution of the QC covariates. Cytoplasmic RNAs are usually lost but mitochondrial RNAs are retained for broken cells, thus barcodes (cells) with a low count depth, few detected genes, and a high fraction of mitochondria counts are indicative of low-quality cells. Cells with unexpectedly high counts and a large number of detected genes may represent doublets, which are artifactual libraries generated from two cells and can lead to spurious biological conclusions. The high-count depth threshold can be used to exclude doublets. In addition, computational doublet detection tools (e.g., DoubletFinder) based on gene expression features can be used [77]. Since there are no specific threshold values for the QC covariates, it may be necessary to revisit quality control decisions multiple times when analyzing the data [78].

### 4.2. Feature Selection and Visualization

Human scRNA-seq data are high dimensional since they measure expression levels of thousands of genes (up to 25,000) in a large number of cells. Feature selection is a key step in many single-cell RNA-seq analyses to keep only genes that are informative of the variability in the single-cell RNA-seq data. Highly Variable Genes (HVGs) method [79], which relies on the assumption that the genes with highly variable expression across cells are resulted from biological effects rather than technical noise, is often used. Typically, between 1000 and 5000 HVGs are selected for downstream analysis. Further, scRNA-seq data visualization can be performed in four main steps: normalization, feature selection (HVGs), dimensionality reduction with principal components analysis (PCA), and projection of scRNA-seq data in an embedded space such as T-distributed Stochastic Neighbor Embedding (t-SNE) and Uniform Manifold Approximation and Projection (UMAP). Typically, PCA identifies the directions of the top N principal components and transforms the data in the low dimension space. Each component is used to infer which genes are contributing the most to variance in the population and are involved in differentiating cells’ subpopulations. The number of N principal components can be determined by “elbow” heuristics or the permutation-test-based jackstraw method. t-SNE is a way of converting a high-dimensional data set into a matrix of pair-wise similarities. This technique reveals the local structure of the high-dimensional data, while also discloses the global structure such as the presence of clusters at several scales [80]. Briefly, the algorithm projects the data points on the 2D plane, initially at random positions, and lets them interact as if they were physical particles. The interaction is governed by two laws: first, all points are repelled from each other; second, each point is attracted to its nearest neighbours, allowing, in this way, the clustering of data points that are actually more similar from the transcriptional point of view [81]. However, t-SNE suffers from several limitations such as loss of the intercluster relationships, slow computation time and weak reproducibility in representing very large datasets. On the contrary, UMAP, was confirmed to provide faster run times, higher reproducibility on big datasets and meaningful organization of cell clusters [82].

#### Cell Type Identification and Population Analysis

A common data analysis pipeline for scRNA-seq data includes the identification of distinct cell types to unravel the cellular heterogeneity of samples. The identification should be carried out after QC and normalization. This task is typically solved by unsupervised clustering methods and manual annotation based on canonical gene markers (e.g., genes associated with a cluster of differentiation (CD) markers in the immune cells). The clustering algorithms specialized for the scRNA-seq data are generally declined from some type of general k-mean, graph-based, density-based, or hierarchical clustering. The cells are grouped into clusters based on the similarity of their gene expression profiles. After clustering, the gene markers are used to characterize and annotate the clusters with a meaningful biological label. Since the manual annotation is a time-consuming process and not reproducible across different experiments within and across research groups, a growing number of classification approaches based on machine learning algorithms and artificial neural networks are being adapted to automatically label cells. Recently, twenty-two automatic cell type identification methods were evaluated using 27 publicly available scRNA-seq data sets of different sizes, technologies, species, and levels of complexity [83]. In the evaluation, general-purpose SVM rejection classifier (with a linear kernel) provided the best performance across all data sets [84].

### 4.3. High-Throughput Transcriptomics

High-throughput transcriptomics technologies represent diverse technical solutions to increase the throughput and reduce the cost of gene expression profiling, and frequently this includes measuring less than the full genomic complement of genes. For descriptions of individual technologies, please refer to the part I in this series of reviews. Preprocessing protocols can also be highly customized and manufacturer specific, e.g., the L1000 technology [5]. In this case, preprocessed data or methods are usually provided by the technology manufacturer or service provider as part of the service fee. But in general if a sizable fraction of the whole transcriptome has been measured, e.g., from a few thousands to the full genome such as with the S1500+ platform [85], the same methods can be used as for microarray or RNA-seq analysis. Continuous data can be treated as microarray data and count-based as RNA-seq data. Global normalization methods, such as RMA and especially LOWESS, can also be used with lower numbers of genes [86]. If the number of measured genes is below the low hundreds or the gene complement is highly biased customized normalization methods are usually employed. These can be similar to the methods used for qPCR arrays, e.g., standardization using house-keeping or other invariant or least variant genes followed by an intensity-based correction [87,88]. High-throughput technologies frequently have higher noise levels than standard established full-genome technologies, so it may be advisable to analyse a larger number of biological replicates e.g., going from the recommended three up to five or more. Although this negates part of the cost-savings, the cost of the profiling per-sample can still be an order of magnitude less than with conventional technologies.

## 5. Differential Expression Analysis

A gene is considered to be differentially expressed if the difference between its expression measured in two experimental conditions is statistically significant. For microarray experiments, differential expression analysis can be performed in R by using the limma package [32]. Limma uses linear models to estimate the covariate dependencies between samples and the variability in the data set. Indeed, the lmFit function from the limma package fits gene-wise linear models to the microarray data. The user defines the design for the model by providing the biological variable of interest and covariates (biological and technical batch variables). Since the variability in the expression matrix can be due both to biological and technical variables, as discussed above, it is important to include as covariates of the limma model the batch effect variables that have been used in the batch removal step. The contrasts of interest are then specified to obtain contrast specific coefficients from the linear model. The eBayes function is applied to assess differential expression by using the fitted model with the contrast coefficients. Furthermore, an adjustment method for the *p*-values is applied to avoid errors given by the multiple testing procedure. The classical approach to control for multiple testing is by familywise error rate (FWER), which focuses on avoiding the Type I errors (‘false positives’) in a very strict way. Examples of methodologies that fall into this category are the Bonferroni method and its variant Holm’s method [89], as well as Hommel [90], and Hochberg methods [91] respectively, with the first two having the advantage that no assumption on the dependence structure of *p*-values is made. However, the aforementioned procedures have been proven to be overconservative for many applications of genomics. Nowadays, the most popular method for multiple testing adjustment is the one developed by Benjamini and Hochberg [92], which controls the false discovery rate (FDR). FDR is much less conservative and addresses the proportion of the ‘false positives’ or ‘false discoveries’ in the selected set of differentially expressed genes, which is advantageous in exploratory genomic analyses [93,94]. Final reporting of the differentially expressed genes is performed by using the toptable function.This analysis gives in output for every gene a fold change, that explains how different is the gene expression value between the conditions, and a *p*-value that explains how significant is that difference. The user may set up a threshold on the *p*-value and fold-change to identify the final set of differentially expressed genes.

The limma package works well also with RNA-Seq experiments, with few adjustments for this data type. Indeed, limma transforms the read counts matrix in log2-counts-per-million (logCPM) and the mean variance relationship can be modelled with two different approaches: precision weights and an empirical Bayes prior trend [95,96]. For the first case, limma implements the function voom, which should be used in case the library sizes of the samples are quite variable, and, optionally, one can apply a between-samples normalization (e.g., quantile). For homogeneous library sizes, the second approach is preferred, and it can be employed through the argument trend in the eBayes function. Similarly, edgeR [59,97] and DESeq2 [60] are two among the most used tools for differential expression analysis. Their implementation and usage is quite similar, if not overlapping to the limma one. The main difference is that they are specifically designed for Next Generation Sequencing analyses, thus taking into account all the characteristics of this kind of data. Briefly, they fit a negative binomial model prior to the multiple testing for differential expression. Both tools take in input unnormalized read counts (although edgeR accepts also normalized counts), and calculate a normalization factor and scale the counts accordingly. Finally, the NOISeq package [98] provides many useful tools in order to prepare the data and perform a differential expression analysis, such as (1) quality control of the read counts, (2) counts normalization and filtering, and (3) multiple testing for differential expression. NOISeq can take in input both normalized or unnormalized data and the applied test is non-parametric, so no assumption on the data distribution is made for the analysis. NOISeq method was optimized to compute differential expression on data with technical replicates. In case of biological replicates, the authors developed the NOISeqBIO algorithm, which is, instead, optimized for this kind of setting. Note that NOISeq also implements the NOISeq-sim algorithm, which can be useful in case no replicates are available. NOISeq-sim simulates technical replicates from a multinomial distribution, however we discourage the users from applying it for testing if the aim is a robust differential expression analysis.

## 6. Gene Functional Annotation and Pathway Analysis

The functional annotation of genes is an essential step to further interpret the results of transcriptomics experiments. This analysis is usually performed in order to characterize the biological role of a certain gene of interest (e.g., differentially expressed genes) in the cell, as well as its activity in one or more molecular processes and its localization in the cell. The main functional annotation databases are the Kyoto Encyclopedia of Genes and Genomes (KEGG) pathways [99], Reactome pathways [100], Gene Ontology terms [101] (GO) and WikiPathways [102]. All these databases collect lists of genes categorized by different criteria. Functional annotation allows the user to study the distribution of sets of genes in the different annotated categories.

Gene overlap or over-representation analysis with the Fisher’s exact test is the most commonly used statistical method. *P*-values represent the difference between the observed and the expected overlaps between the differentially expressed genes in the experiment and the functional gene set, as well as the numbers of genes involved. Multiple testing correction is performed to control for spurious results. However, as the Fisher’s exact test assumes that differential expression of one gene does not depend on the others (no inter-gene correlations), the test can give nonspecific results with long gene lists. Variants of the classical Fisher’s exact test implemented in e.g., the topGO-package can mitigate nonspecific results from large gene sets at the top of the GO-hierarchy by taking advantage of the dependencies in the directed-acyclic graph structure of the database [103].

Gene Set Enrichment Analysis (GSEA), which is also referred to as functional class scoring, is a rank-based threshold-free method that does not rely on differentially expressed genes to perform pathway analyses but uses all available gene expression information [104,105,106]. The benefits of the method include the fact that it operates at pathway level and thus, considers biological complexity, by allowing inclusion of low-level changes that may not be detected in traditional analyses aimed at identifying differentially expressed genes. The method avoids the use of arbitrary fold-change and *p*-value limits, which may be one of the biggest obstacles towards implementation of standardized bioinformatics pipelines in toxicogenomic approaches [107]. Large numbers of GSEA methods exist that give somewhat different results and test for different hypotheses [108]. To avoid nonspecific results, methods that incorporate sample permutation or rotation are used, such as the limma ROAST, ROMER or the Broad Institute GSEA method with sample permutations [32,104]. Large numbers of permutations or rotations may be needed as the minimum two-sided *p*-value is 1/(nrot+1), where nrot is the number of the permutation or rotation steps (*p*-values of zero are especially detrimental). Parametric methods can also be used and may have higher sensitivity, as long as they consider inter-gene correlations, such as the limma CAMERA method [32]. Overall, for smaller-scale analyses, the Broad Institute GSEA tool is a good choice. Annotating results from omics experiments into functional categories is essential not only to understand the underlying regulatory dynamics but also to compare multiple experimental conditions at a higher level of abstraction.

Programmatic larger-scale analyses can be performed with the methods in the limma R package. R and Cytoscape-based workflows can also combine visualizations with pathway analyses in a very powerful manner [109,110]. Fisher’s exact test based methods can be used if there are no other alternatives, especially for GO analyses, although packages such as the topGO are preferable in that case. Furthermore, a multitude of tools are available to the community to graphically represent enriched functional annotations from single pairwise comparisons such as g:Profiler [111], DAVID [112], ToppGene Suite [113], Enrichr [114]. These tools have a nice graphical interface to visualize the results, but they allow the user to analyze one experiment at a time, while comparisons between different runs have to be performed manually. Some other tools have been proposed that are able to compare different experimental conditions at the same time, such as clusterProfiler [115] and BACA [116]. However, they require a certain programming ability in order to produce the desired visualizations. Thus, using these tools is quite complex as the number of experiments to compare increases.

Since more and more toxicogenomics studies involve the comparison of the effect of different materials at the same time, we recently proposed a graphical tool implemented in R, called FunMappOne [110], that enables the users to graphically inspect, navigate, and compare functional annotations in multiple experiments at different levels of abstraction. This tool facilitates the analyses of multiple experimental conditions through a simple user interface and dynamic graphical representations of the relevant functional categories.

## 7. Conclusions

The newly flourished TGx field is lacking rational guidelines for the preprocessing of large scale data deriving from transcriptomics experiments. Addressing this drawback is crucial in order to carry out reliable risk assessment and toxicity prediction. A proper setting of the data preprocessing assures a robust outcome from the downstream analysis, and, in turn, allows to correctly answer the biological questions posed by the study. For this reason, it is important to highlight that the outcome of the analysis, such as the degree of toxicity of a chemical, often depends on thresholds or parameters of algorithms set in all the analytical steps. Thus, particular attention must be paid to these aspects to perform a thoughtful final decision.

In this review, which is the second part of a three papers series, we outlined the best practices in the preprocessing of transcriptomics data derived from DNA microarray, bulk RNA-Seq and scRNA-Seq technologies, and we make them available to the TGx community. Since the batch effect evaluation step is not often considered in the routine analytical practice, we pointed out the most widespread methods and procedures to evaluate and, eventually, correct the data from technical variability. Moreover, we covered the golden standard methods to perform basic steps of the downstream analysis, including differential expression analysis and gene functional annotation. In conclusion, this review article represents a reference survey of “good practices” for transcriptomics data analysis in TGx.

## Supplementary Materials

The following are available online at https://www.mdpi.com/2079-4991/10/5/903/s1, Table S1: CRAN and Bioconductor packages for microarray data analysis; Table S2: Diagnostic methods for microarrays quality check; Table S3: Tools for RNA-Seq data analysis.

## Abbreviations

The following abbreviations are used in this manuscript:

AGA	Automated Genomics Analysis
BACA	Bubble Chart to Compare Biological Annotations
BWT	Burrow-Wheeler Transform
CAMERA	Correlation Adjusted MEan RAnk gene set test
CD	Cluster of Differentiation
CDF	Chip Description File
CEL-Seq	Cell Expression by Linear amplification and Sequencing
CMAP	Connectivity Map
DAVID	Database for Annotation, Visualization and Integrated Discovery
Drop-seq	Droplet sequencing
ENMs	Engineered Nanomaterials
eUTOPIA	solUTion for Omics data PreprocessIng and Analysis
FDR	False Discovery Rate
FPKM	Fragments Per Kilobase of exon model per Million mapped reads
FWER	Family-Wise Error Rate
GEO	Gene Expression Omnibus
GFM	Graph-based FM index
GLM	Generalized Linear Models
GO	Gene Ontology
GSEA	Gene Set Enrichment Analysis
HISAT2	Hierarchical Indexing for Spliced Alignment of Transcripts 2
HVGs	Highly Variable Genes
KBET	K-nearest neighbor Batch Effect Test
KEGG	Kyoto Encyclopedia of Genes and Genomes
L1000	Library of Integrated Network-based Cellular Signatures 1000
logCPM	log2-Counts Per Million
LOWESS	LOcally WEighted Scatterplot Smoothing
MARS-seq	MAssively parallel single-cell RNA-Sequencing
MeV	MultiExperiment Viewer
MNN	Mutual Nearest Neighbor
NCBI	National Center for Biotechnology Information
NGS	Next Generation Sequencing
Open TG-GATEs	ToxicoGenomics project-Genomics Assisted Toxicity Evaluation system
PCA	Principal Component Analysis
QC	Quality Check
RefSeq	Reference Sequence collection
RIN	RNA Integrity Number
RNA-seq	RNA sequencing
ROAST	ROtAtion Gene Set Tests
ROMER	ROtation testing using MEan Ranks
RPKM	Reads Per Kilobase of exon model per Million mapped reads
RSEM	RNA-Seq by Expectation Maximization
SAVER	Single-cell Analysis Via Expression Recovery
scRNA-seq	single cell RNA sequencing
SEQC	Sequence Quality Control
SVA	Surrogate Variable Analysis
SVM	Support Vector Machine
TGx	ToxicoGenomics
t-SNE	t-distributed Stochastic Neighbor Embedding
UMAP	Uniform Manifold Approximation and Projection
UMI	Unique Molecular Identifiers
UQUA	Upper Quantile

## References

[1] Waters M.D., Fostel J.M. (2004). Toxicogenomics and systems toxicology: Aims and prospects. Nat. Rev. Genet..

[2] Alexander-Dann B., Pruteanu L.L., Oerton E., Sharma N., Berindan-Neagoe I., Módos D., Bender A. (2018). Developments in toxicogenomics: Understanding and predicting compound-induced toxicity from gene expression data. Mol. Omics.

[3] Casamassimi A., Federico A., Rienzo M., Esposito S., Ciccodicola A. (2017). Transcriptome profiling in human diseases: New advances and perspectives. Int. J. Mol. Sci..

[4] Lamb J. (2007). The Connectivity Map: A new tool for biomedical research. Nat. Rev. Cancer.

[5] Subramanian A., Narayan R., Corsello S.M., Peck D.D., Natoli T.E., Lu X., Gould J., Davis J.F., Tubelli A.A., Asiedu J.K. (2017). A next generation connectivity map: L1000 platform and the first 1,000,000 profiles. Cell.

[6] Ganter B., Snyder R.D., Halbert D.N., Lee M.D. (2006). Toxicogenomics in drug discovery and development: Mechanistic analysis of compound/class-dependent effects using the DrugMatrix® database. Future Med..

[7] Igarashi Y., Nakatsu N., Yamashita T., Ono A., Ohno Y., Urushidani T., Yamada H. (2015). Open TG-GATEs: A large-scale toxicogenomics database. Nucleic Acids Res..

[8] Kolesnikov N., Hastings E., Keays M., Melnichuk O., Tang Y.A., Williams E., Dylag M., Kurbatova N., Brandizi M., Burdett T. (2015). ArrayExpress update—Simplifying data submissions. Nucleic Acids Res..

[9] Edgar R., Domrachev M., Lash A.E. (2002). Gene Expression Omnibus: NCBI gene expression and hybridization array data repository. Nucleic Acids Res..

[10] Conesa A., Madrigal P., Tarazona S., Gomez-Cabrero D., Cervera A., McPherson A., Szcześniak M.W., Gaffney D.J., Elo L.L., Zhang X. (2016). A survey of best practices for RNA-seq data analysis. Genome Biol..

[11] Oshlack A., Robinson M.D., Young M.D. (2010). From RNA-seq reads to differential expression results. Genome Biol..

[12] Witten D.M., Tibshirani R. (2013). Scientific research in the age of omics: The good, the bad, and the sloppy. J. Am. Med. Inform. Assoc..

[13] Russo F., Righelli D., Angelini C. (2015). Advantages and limits in the adoption of reproducible research and R-tools for the analysis of omic data. International Meeting on Computational Intelligence Methods for Bioinformatics and Biostatistics.

[14] Quackenbush J. (2002). Microarray data normalization and transformation. Nat. Genet..

[15] Allison D.B., Cui X., Page G.P., Sabripour M. (2006). Microarray data analysis: From disarray to consolidation and consensus. Nat. Rev. Genet..

[16] Lee E.K., Park T. (2007). Exploratory methods for checking quality of microarray data. Bioinformation.

[17] Bolstad B.M., Collin F., Simpson K.M., Irizarry R.A., Speed T.P. (2004). Experimental design and low-level analysis of microarray data. Int. Rev. Neurobiol..

[18] Fasold M., Binder H. (2013). AffyRNADegradation: Control and correction of RNA quality effects in GeneChip expression data. Bioinformatics.

[19] Eijssen L.M., Jaillard M., Adriaens M.E., Gaj S., de Groot P.J., Müller M., Evelo C.T. (2013). User-friendly solutions for microarray quality control and pre-processing on ArrayAnalysis. org. Nucleic Acids Res..

[20] Gavin A.J.S. (2016). Investigating the Mechanisms of Silver Nanoparticle Toxicity in Daphnia Magna: A Multi-Omics Approach. Ph.D. Thesis.

[21] Yang Y.H., Dudoit S., Luu P., Lin D.M., Peng V., Ngai J., Speed T.P. (2002). Normalization for cDNA microarray data: A robust composite method addressing single and multiple slide systematic variation. Nucleic Acids Res..

[22] Bilban M., Buehler L.K., Head S., Desoye G., Quaranta V. (2002). Normalizing DNA microarray data. Curr. Issues Mol. Biol..

[23] Yang Y.H., Dudoit S., Luu P., Speed T.P. (2001). Normalization for cDNA microarry data. Microarrays: Optical Technologies and Informatics.

[24] Cleveland W.S. (1979). Robust locally weighted regression and smoothing scatterplots. J. Am. Stat. Assoc..

[25] Hicks S.C., Irizarry R.A. (2015). Quantro: A data-driven approach to guide the choice of an appropriate normalization method. Genome Biol..

[26] Irizarry R.A., Bolstad B.M., Collin F., Cope L.M., Hobbs B., Speed T.P. (2003). Summaries of Affymetrix GeneChip probe level data. Nucleic Acids Res..

[27] Kupfer P., Guthke R., Pohlers D., Huber R., Koczan D., Kinne R.W. (2012). Batch correction of microarray data substantially improves the identification of genes differentially expressed in rheumatoid arthritis and osteoarthritis. BMC Med. Genom..

[28] Lazar C., Meganck S., Taminau J., Steenhoff D., Coletta A., Molter C., Weiss-Solís D.Y., Duque R., Bersini H., Nowé A. (2013). Batch effect removal methods for microarray gene expression data integration: A survey. Briefings Bioinform..

[29] Leek J.T., Johnson W.E., Parker H.S., Jaffe A.E., Storey J.D. (2012). The sva package for removing batch effects and other unwanted variation in high-throughput experiments. Bioinformatics.

[30] Leek J.T., Scharpf R.B., Bravo H.C., Simcha D., Langmead B., Johnson W.E., Geman D., Baggerly K., Irizarry R.A. (2010). Tackling the widespread and critical impact of batch effects in high-throughput data. Nat. Rev. Genet..

[31] Johnson W.E., Li C., Rabinovic A. (2007). Adjusting batch effects in microarray expression data using empirical Bayes methods. Biostatistics.

[32] Ritchie M.E., Phipson B., Wu D., Hu Y., Law C.W., Shi W., Smyth G.K. (2015). limma powers differential expression analyses for RNA-sequencing and microarray studies. Nucleic Acids Res..

[33] Arloth J., Bader D.M., Röh S., Altmann A. (2015). Re-Annotator: Annotation pipeline for microarray probe sequences. PLoS ONE.

[34] Huber W., Carey V.J., Gentleman R., Anders S., Carlson M., Carvalho B.S., Bravo H.C., Davis S., Gatto L., Girke T. (2015). Orchestrating high-throughput genomic analysis with Bioconductor. Nat. Methods.

[35] Considine M., Parker H., Wei Y., Xia X., Cope L., Ochs M., Fertig E. (2015). AGA: Interactive pipeline for reproducible gene expression and DNA methylation data analyses. F1000Research.

[36] Howe E.A., Sinha R., Schlauch D., Quackenbush J. (2011). RNA-Seq analysis in MeV. Bioinformatics.

[37] Cutts R.J., Dayem Ullah A.Z., Sangaralingam A., Gadaleta E., Lemoine N.R., Chelala C. (2012). O-miner: An integrative platform for automated analysis and mining of-omics data. Nucleic Acids Res..

[38] Kallio M.A., Tuimala J.T., Hupponen T., Klemelä P., Gentile M., Scheinin I., Koski M., Käki J., Korpelainen E.I. (2011). Chipster: User-friendly analysis software for microarray and other high-throughput data. BMC Genom..

[39] Alonso R., Salavert F., Garcia-Garcia F., Carbonell-Caballero J., Bleda M., Garcia-Alonso L., Sanchis-Juan A., Perez-Gil D., Marin-Garcia P., Sanchez R. (2015). Babelomics 5.0: Functional interpretation for new generations of genomic data. Nucleic Acids Res..

[40] Marwah V.S., Scala G., Kinaret P.A.S., Serra A., Alenius H., Fortino V., Greco D. (2019). eUTOPIA: solUTion for Omics data PreprocessIng and Analysis. Source Code Biol. Med..

[41] Auer H., Lyianarachchi S., Newsom D., Klisovic M.I., Marcucci G., Marcucci U., Kornacker K. (2003). Chipping away at the chip bias: RNA degradation in microarray analysis. Nat. Genet..

[42] Schroeder A., Mueller O., Stocker S., Salowsky R., Leiber M., Gassmann M., Lightfoot S., Menzel W., Granzow M., Ragg T. (2006). The RIN: An RNA integrity number for assigning integrity values to RNA measurements. BMC Mol. Biol..

[43] Gallego Romero I., Pai A.A., Tung J., Gilad Y. (2014). RNA-seq: Impact of RNA degradation on transcript quantification. BMC Biol..

[44] Ewing B., Green P. (1998). Base-calling of automated sequencer traces using phred. II. Error probabilities. Genome Res..

[45] Martin M. (2011). Cutadapt removes adapter sequences from high-throughput sequencing reads. EMBnet. J..

[46] Trapnell C., Pachter L., Salzberg S.L. (2009). TopHat: Discovering splice junctions with RNA-Seq. Bioinformatics.

[47] Ameur A., Wetterbom A., Feuk L., Gyllensten U. (2010). Global and unbiased detection of splice junctions from RNA-seq data. Genome Biol..

[48] Kim D., Pertea G., Trapnell C., Pimentel H., Kelley R., Salzberg S.L. (2013). TopHat2: Accurate alignment of transcriptomes in the presence of insertions, deletions and gene fusions. Genome Biol..

[49] Pertea M., Kim D., Pertea G.M., Leek J.T., Salzberg S.L. (2016). Transcript-level expression analysis of RNA-seq experiments with HISAT, StringTie and Ballgown. Nat. Protoc..

[50] Roberts A., Pimentel H., Trapnell C., Pachter L. (2011). Identification of novel transcripts in annotated genomes using RNA-Seq. Bioinformatics.

[51] Pruitt K.D., Tatusova T., Maglott D.R. (2007). NCBI reference sequences (RefSeq): A curated non-redundant sequence database of genomes, transcripts and proteins. Nucleic Acids Res..

[52] Wilming L.G., Gilbert J.G., Howe K., Trevanion S., Hubbard T., Harrow J.L. (2007). The vertebrate genome annotation (Vega) database. Nucleic Acids Res..

[53] Liao Y., Smyth G.K., Shi W. (2019). The R package Rsubread is easier, faster, cheaper and better for alignment and quantification of RNA sequencing reads. Nucleic Acids Res..

[54] Anders S., Pyl P.T., Huber W. (2015). HTSeq—A Python framework to work with high-throughput sequencing data. Bioinformatics.

[55] Mortazavi A., Williams B.A., McCue K., Schaeffer L., Wold B. (2008). Mapping and quantifying mammalian transcriptomes by RNA-Seq. Nat. Methods.

[56] Evans C., Hardin J., Stoebel D.M. (2018). Selecting between-sample RNA-Seq normalization methods from the perspective of their assumptions. Briefings Bioinform..

[57] Bullard J.H., Purdom E., Hansen K.D., Dudoit S. (2010). Evaluation of statistical methods for normalization and differential expression in mRNA-Seq experiments. BMC Bioinform..

[58] Bourgon R., Gentleman R., Huber W. (2010). Independent filtering increases detection power for high-throughput experiments. Proc. Natl. Acad. Sci. USA.

[59] Robinson M.D., McCarthy D.J., Smyth G.K. (2010). edgeR: A Bioconductor package for differential expression analysis of digital gene expression data. Bioinformatics.

[60] Love M.I., Huber W., Anders S. (2014). Moderated estimation of fold change and dispersion for RNA-seq data with DESeq2. Genome Biol..

[61] Risso D., Ngai J., Speed T.P., Dudoit S. (2014). Normalization of RNA-seq data using factor analysis of control genes or samples. Nat. Biotechnol..

[62] Gagnon-Bartsch J.A., Speed T.P. (2012). Using control genes to correct for unwanted variation in microarray data. Biostatistics.

[63] Jacob L., Gagnon-Bartsch J.A., Speed T.P. (2016). Correcting gene expression data when neither the unwanted variation nor the factor of interest are observed. Biostatistics.

[64] Gagnon-Bartsch J.A., Jacob L., Speed T.P. (2013). Removing Unwanted Variation from High Dimensional Data with Negative Controls.

[65] Tang F., Barbacioru C., Wang Y., Nordman E., Lee C., Xu N., Wang X., Bodeau J., Tuch B.B., Siddiqui A. (2009). mRNA-Seq whole-transcriptome analysis of a single cell. Nat. Methods.

[66] Rostom R., Svensson V., Teichmann S.A., Kar G. (2017). Computational approaches for interpreting scRNA-seq data. FEBS Lett..

[67] Zappia L., Phipson B., Oshlack A. (2018). Exploring the single-cell RNA-seq analysis landscape with the scRNA-tools database. PLoS Comput. Biol..

[68] Zheng G.X., Terry J.M., Belgrader P., Ryvkin P., Bent Z.W., Wilson R., Ziraldo S.B., Wheeler T.D., McDermott G.P., Zhu J. (2017). Massively parallel digital transcriptional profiling of single cells. Nat. Commun..

[69] Klein A.M., Mazutis L., Akartuna I., Tallapragada N., Veres A., Li V., Peshkin L., Weitz D.A., Kirschner M.W. (2015). Droplet barcoding for single-cell transcriptomics applied to embryonic stem cells. Cell.

[70] Azizi E., Carr A.J., Plitas G., Cornish A.E., Konopacki C., Prabhakaran S., Nainys J., Wu K., Kiseliovas V., Setty M. (2018). Single-cell map of diverse immune phenotypes in the breast tumor microenvironment. Cell.

[71] Parekh S., Ziegenhain C., Vieth B., Enard W., Hellmann I. (2018). zUMIs-a fast and flexible pipeline to process RNA sequencing data with UMIs. Gigascience.

[72] Bacher R., Chu L.F., Leng N., Gasch A.P., Thomson J.A., Stewart R.M., Newton M., Kendziorski C. (2017). SCnorm: Robust normalization of single-cell RNA-seq data. Nat. Methods.

[73] Lun A.T., Bach K., Marioni J.C. (2016). Pooling across cells to normalize single-cell RNA sequencing data with many zero counts. Genome Biol..

[74] Büttner M., Miao Z., Wolf F.A., Teichmann S.A., Theis F.J. (2019). A test metric for assessing single-cell RNA-seq batch correction. Nat. Methods.

[75] Ilicic T., Kim J.K., Kolodziejczyk A.A., Bagger F.O., McCarthy D.J., Marioni J.C., Teichmann S.A. (2016). Classification of low quality cells from single-cell RNA-seq data. Genome Biol..

[76] Griffiths J.A., Scialdone A., Marioni J.C. (2018). Using single-cell genomics to understand developmental processes and cell fate decisions. Mol. Syst. Biol..

[77] McGinnis C.S., Murrow L.M., Gartner Z.J. (2019). DoubletFinder: Doublet detection in single-cell RNA sequencing data using artificial nearest neighbors. Cell Syst..

[78] Luecken M.D., Theis F.J. (2019). Current best practices in single-cell RNA-seq analysis: A tutorial. Mol. Syst. Biol..

[79] Brennecke P., Anders S., Kim J.K., Kołodziejczyk A.A., Zhang X., Proserpio V., Baying B., Benes V., Teichmann S.A., Marioni J.C. (2013). Accounting for technical noise in single-cell RNA-seq experiments. Nat. Methods.

[80] Maaten L., Hinton G. (2008). Visualizing data using t-SNE. J. Mach. Learn. Res..

[81] Kobak D., Berens P. (2019). The art of using t-SNE for single-cell transcriptomics. Nat. Commun..

[82] Becht E., McInnes L., Healy J., Dutertre C.A., Kwok I.W., Ng L.G., Ginhoux F., Newell E.W. (2019). Dimensionality reduction for visualizing single-cell data using UMAP. Nat. Biotechnol..

[83] Abdelaal T., Michielsen L., Cats D., Hoogduin D., Mei H., Reinders M.J., Mahfouz A. (2019). A comparison of automatic cell identification methods for single-cell RNA sequencing data. Genome Biol..

[84] Pedregosa F., Varoquaux G., Gramfort A., Michel V., Thirion B., Grisel O., Blondel M., Prettenhofer P., Weiss R., Dubourg V. (2011). Scikit-learn: Machine learning in Python. J. Mach. Learn. Res..

[85] Yeakley J.M., Shepard P.J., Goyena D.E., VanSteenhouse H.C., McComb J.D., Seligmann B.E. (2017). A trichostatin A expression signature identified by TempO-Seq targeted whole transcriptome profiling. PLoS ONE.

[86] Mar J.C., Kimura Y., Schroder K., Irvine K.M., Hayashizaki Y., Suzuki H., Hume D., Quackenbush J. (2009). Data-driven normalization strategies for high-throughput quantitative RT-PCR. BMC Bioinform..

[87] Calza S., Valentini D., Pawitan Y. (2008). Normalization of oligonucleotide arrays based on the least-variant set of genes. BMC Bioinform..

[88] Cui X., Yu S., Tamhane A., Causey Z.L., Steg A., Danila M.I., Reynolds R.J., Wang J., Wanzeck K.C., Tang Q. (2015). Simple regression for correcting ΔC t bias in RT-qPCR low-density array data normalization. BMC Genom..

[89] Holm S. (1979). A simple sequentially rejective multiple test procedure. Scand. J. Stat..

[90] Hommel G. (1988). A stagewise rejective multiple test procedure based on a modified Bonferroni test. Biometrika.

[91] Hochberg Y. (1988). A sharper Bonferroni procedure for multiple tests of significance. Biometrika.

[92] Benjamini Y., Hochberg Y. (1995). Controlling the false discovery rate: A practical and powerful approach to multiple testing. J. R. Stat. Soc. Ser. B (Methodol.).

[93] Goeman J.J., Solari A. (2014). Multiple hypothesis testing in genomics. Stat. Med..

[94] Benjamini Y., Yekutieli D. (2001). The control of the false discovery rate in multiple testing under dependency. Ann. Stat..

[95] Law C.W., Chen Y., Shi W., Smyth G.K. (2014). voom: Precision weights unlock linear model analysis tools for RNA-seq read counts. Genome Biol..

[96] Liu R., Holik A.Z., Su S., Jansz N., Chen K., Leong H.S., Blewitt M.E., Asselin-Labat M.L., Smyth G.K., Ritchie M.E. (2015). Why weight? Modelling sample and observational level variability improves power in RNA-seq analyses. Nucleic Acids Res..

[97] McCarthy D.J., Chen Y., Smyth G.K. (2012). Differential expression analysis of multifactor RNA-Seq experiments with respect to biological variation. Nucleic Acids Res..

[98] Tarazona S., Furió-Tarí P., Turrà D., Pietro A.D., Nueda M.J., Ferrer A., Conesa A. (2015). Data quality aware analysis of differential expression in RNA-seq with NOISeq R/Bioc package. Nucleic Acids Res..

[99] Kanehisa M., Sato Y., Kawashima M., Furumichi M., Tanabe M. (2016). KEGG as a reference resource for gene and protein annotation. Nucleic Acids Res..

[100] Croft D., Mundo A.F., Haw R., Milacic M., Weiser J., Wu G., Caudy M., Garapati P., Gillespie M., Kamdar M.R. (2014). The Reactome pathway knowledgebase. Nucleic Acids Res..

[101] Ashburner M., Ball C.A., Blake J.A., Botstein D., Butler H., Cherry J.M., Davis A.P., Dolinski K., Dwight S.S., Eppig J.T. (2000). Gene ontology: Tool for the unification of biology. Nat. Genet..

[102] Slenter D.N., Kutmon M., Hanspers K., Riutta A., Windsor J., Nunes N., Mélius J., Cirillo E., Coort S.L., Digles D. (2018). WikiPathways: A multifaceted pathway database bridging metabolomics to other omics research. Nucleic Acids Res..

[103] Alexa A., Rahnenführer J., Lengauer T. (2006). Improved scoring of functional groups from gene expression data by decorrelating GO graph structure. Bioinformatics.

[104] Subramanian A., Tamayo P., Mootha V.K., Mukherjee S., Ebert B.L., Gillette M.A., Paulovich A., Pomeroy S.L., Golub T.R., Lander E.S. (2005). Gene set enrichment analysis: A knowledge-based approach for interpreting genome-wide expression profiles. Proc. Natl. Acad. Sci. USA.

[105] Khatri P., Sirota M., Butte A.J. (2012). Ten years of pathway analysis: Current approaches and outstanding challenges. PLoS Comput. Biol..

[106] Grafström R.C., Nymark P., Hongisto V., Spjuth O., Ceder R., Willighagen E., Hardy B., Kaski S., Kohonen P. (2015). Toward the replacement of animal experiments through the bioinformatics-driven analysis of ‘omics’ data from human cell cultures. Altern. Lab. Anim..

[107] Dean J.L., Zhao Q.J., Lambert J.C., Hawkins B.S., Thomas R.S., Wesselkamper S.C. (2017). Application of Gene Set Enrichment Analysis for Identification of Chemically-Induced, Biologically Relevant Transcriptomic Networks and Potential Utilization in Human Health Risk Assessment. Toxicol. Sci..

[108] Rahmatallah Y., Emmert-Streib F., Glazko G. (2016). Gene set analysis approaches for RNA-seq data: Performance evaluation and application guideline. Briefings Bioinform..

[109] Reimand J., Isserlin R., Voisin V., Kucera M., Tannus-Lopes C., Rostamianfar A., Wadi L., Meyer M., Wong J., Xu C. (2019). Pathway enrichment analysis and visualization of omics data using g: Profiler, GSEA, Cytoscape and EnrichmentMap. Nat. Protoc..

[110] Scala G., Serra A., Marwah V.S., Saarimäki L.A., Greco D. (2019). FunMappOne: A tool to hierarchically organize and visually navigate functional gene annotations in multiple experiments. BMC Bioinform..

[111] Reimand J., Kull M., Peterson H., Hansen J., Vilo J. (2007). g: Profiler—A web-based toolset for functional profiling of gene lists from large-scale experiments. Nucleic Acids Res..

[112] Huang D.W., Sherman B.T., Lempicki R.A. (2009). Systematic and integrative analysis of large gene lists using DAVID bioinformatics resources. Nat. Protoc..

[113] Chen J., Bardes E.E., Aronow B.J., Jegga A.G. (2009). ToppGene Suite for gene list enrichment analysis and candidate gene prioritization. Nucleic Acids Res..

[114] Kuleshov M.V., Jones M.R., Rouillard A.D., Fernandez N.F., Duan Q., Wang Z., Koplev S., Jenkins S.L., Jagodnik K.M., Lachmann A. (2016). Enrichr: A comprehensive gene set enrichment analysis web server 2016 update. Nucleic Acids Res..

[115] Yu G., Wang L.G., Han Y., He Q.Y. (2012). clusterProfiler: An R package for comparing biological themes among gene clusters. Omics J. Integr. Biol..

[116] Fortino V., Alenius H., Greco D. (2015). BACA: Bubble chArt to compare annotations. BMC Bioinform..

