# Supplementary material for: Transcriptomics in Toxicogenomics, Part II: Preprocessing and Differential Expression Analysis for High Quality Data
Source: Nanomaterials (Basel). 2020 May 8;10(5):903. doi: 10.3390/nano10050903 (PMC7279140; doi:10.3390/nano10050903)
Supplement: Supplementary file 1 [file nanomaterials-10-00903-s001.zip › Table_S2_PartII.pdf]

Diagnostic methods for microarrays quality check

**Category**	**Plot**	**R/Bioconductor Packages**
Sample Quality	Sample prep controls	simpleaffy
	3’/5’ for b’actin and GAPDH	simpleaffy
	RNA degradation plot	affy
Hybridization and overall signal quality	Spike-in hybrid controls	simpleaffy
	Background intensity	simpleaffy
	Percentage present	simpleaffy
	Preset/Marg./Absent calls	simpleaffy
	Pos/Neg control distribution	AffyQCReport
	All affymetrix controls	affy, ArrayTools
Signal comparability and bias diagnostic	Scale factors	simpleaffy
	Boxplot of log-intensity	affy
	Density histogram	affy
	MA plot	affy
	Array reference layout	affy
	Pos/Neg controls COI plot	affyQCReport
	2D images	affy, affyPLM
	NUSE plot	affyPLM
	RLE plot	affyPLM
Array correlation	Correlation plot	affy, gplots
	Hierarchical clustering dendrogram	affy, bioDist
	PCA plot	affy
Summary	Summary table	simpleaffy
