# Supplementary material for: Transcriptomics in Toxicogenomics, Part II: Preprocessing and Differential Expression Analysis for High Quality Data
Source: Nanomaterials (Basel). 2020 May 8;10(5):903. doi: 10.3390/nano10050903 (PMC7279140; doi:10.3390/nano10050903)
Supplement: Supplementary file 1 [file nanomaterials-10-00903-s001.zip › Table_S1_PartII.pdf]

CRAN and Bioconductor packages for Microarray data analysis

**Analysis Step**	**Agilent 2-color**	**Agilent 1-color**	**Affymetrix**	**Illumina Methylation**
Raw Data	limma::read.maimages	limma::read.maimages	affy::justRMA	minfi::read.metharray.exp
QC	arrayQualityMetrics::arrayQuality Metrics	arrayQualityMetrics::arrayQuality Metrics	yaqcaffy::yaqc	shinyMethyl
Probe Quality Estimation	Custom function(); by using signal distribution of negative control probes	Custom function(); by using signal distribution of negative control probes	NA	minfi::detectionP
Sample and/or Array Variation Diagnostics	limma::plotDensities, limma::plotMD, boxplot	limma::plotDensities, boxplot	limma::plotDensities, boxplot	minfi::densityPlot, boxplot
Normalization	limma::normalizeBetweenArrays, limma::normalizeQuantiles, limma::normalizeVSN, limma::normalizeCyclicLoess	limma::normalizeQuantiles, limma::normalizeVSN, limma::normalizeCyclicLoess, limma::normalizeBetweenArrays	Handled by justRMA (Raw Data)	minfi::preprocessRaw, minfi::preprocessSWAN, minfi::preprocessRaw, minfi::preprocessQuantile, minfi::preprocessIllumina, minfi::preprocessFunnorm, minfi::preprocessNoob

Analysis Step		Agilent 2-color	Agilent 1-color	Affymetrix	Illumina Methylation
Batch Effect Estimation		swamp::confounding, swamp::prince, swamp::prince.plot, limma:::plotMDS	swamp::confounding, swamp::prince, swamp::prince.plot, limma:::plotMDS	swamp::confounding, swamp::prince, swamp::prince.plot, limma:::plotMDS	swamp::confounding, swamp::prince, swamp::prince.plot, limma:::plotMDS
Batch Effect Mitigation	Known	Custom function(); employs sva::ComBat to iteratively isolate and correct each batch	Custom function(); employs sva::ComBat to iteratively isolate and correct each batch	Custom function(); employs sva::ComBat to iteratively isolate and correct each batch	Custom function(); employs sva::ComBat to iteratively isolate and correct each batch
	Unknown	1. sva::sva for identification of unknown sources of variation; 2. Estimation of surrogate vairable effect via diagnotic plots; 3. Batch surrogate vairables correction by sva::ComBat	1. sva::sva for identification of unknown sources of variation; 2. Estimation of surrogate vairable effect via diagnotic plots; 3. Batch surrogate vairables correction by sva::ComBat	1. sva::sva for identification of unknown sources of variation; 2. Estimation of surrogate vairable effect via diagnotic plots; 3. Batch surrogate vairables correction by sva::ComBat	1. sva::sva for identification of unknown sources of variation; 2. Estimation of surrogate vairable effect via diagnotic plots; 3. Batch surrogate vairables correction by sva::ComBat

Analysis Step	Agilent 2-color	Agilent 1-color	Affymetrix	Illumina Methylation
Annotation	Array design specific annotation from eArray OR intrinsic annotation from raw data	Array design specific annotation from eArray OR intrinsic annotation from raw data	Array design specific annotation from brainarray; Installable from user interface	IlluminaHumanMethylation450kanno.ilmn12.hg19, IlluminaHumanMethylationEPICanno.ilm10b2.hg19 (from Bioconductor)
Differential Analysis	limma::makeContrasts, limma::lmFit, limma::eBayes, limma::topTable	limma::makeContrasts, limma::lmFit, limma::eBayes, limma::topTable	limma::makeContrasts, limma::lmFit, limma::eBayes, limma::topTable	limma::makeContrasts, limma::lmFit, limma::eBayes, limma::topTable
Data Exploration	1. Differential sets intersection VennDiagram::venn.diagram, UpSetR::upset; 2. Volcano plot via ggplot2::ggplot; 3. Expression values inspection vi multi-gene/multi-probe, multi-sample/group facteted ggplot2::ggplot; 4. Highly differential gene set heatmap via made4::made4::heatplot	1. Differential sets intersection VennDiagram::venn.diagram, UpSetR::upset; 2. Volcano plot via ggplot2::ggplot; 3. Expression values inspection vi multi-gene/multi-probe, multi-sample/group facteted ggplot2::ggplot; 4. Highly differential gene set heatmap via made4::made4::heatplot	1. Differential sets intersection VennDiagram::venn.diagram, UpSetR::upset; 2. Volcano plot via ggplot2::ggplot; 3. Expression values inspection vi multi-gene/multi-probe, multi-sample/group facteted ggplot2::ggplot; 4. Highly differential gene set heatmap via made4::made4::heatplot	1. Differential sets intersection VennDiagram::venn.diagram, UpSetR::upset; 2. Volcano plot via ggplot2::ggplot; 3. Expression values inspection vi multi-gene/multi-probe, multi-sample/group facteted ggplot2::ggplot; 4. Highly differential gene set heatmap via made4::made4::heatplot
