# Supplementary material for: Transcriptomics in Toxicogenomics, Part II: Preprocessing and Differential Expression Analysis for High Quality Data
Source: Nanomaterials (Basel). 2020 May 8;10(5):903. doi: 10.3390/nano10050903 (PMC7279140; doi:10.3390/nano10050903)
Supplement: Supplementary file 1 [file nanomaterials-10-00903-s001.zip › Table_S3_PartII.pdf]

**Analysis Step**	**RNASeq Tools**
Quality Check	FastQC (Standalone)
Alignment	TopHat2 (bash)<br>Hisat2 (bash)
Read counts	HTSeq (Python),<br>Rsubread::featureCounts (R)
Normalization and filtering	Noiseq::UQUA (R),<br>Noiseq::RPKM (R)
Diagnostic plots	Boxplot<br>PCA<br>MDS
Batch effect estimation	swamp::confounding,<br>swamp::prince,<br>swamp::prince.plot,<br>limma:::plotMDS
Batch effect mitigation	sva::ComBat<br>RUVSeq::RUVg - RUVs - RUVr
Differential Expression	edgeR::exactTest<br>edgeR::glmFit<br>Deseq2::DESeq<br>NOISeq::noiseq - noiseqbio
